# Promising Therapy for Heart Failure in Patients with Severe COVID-19: Calming the Cytokine Storm

**DOI:** 10.1007/s10557-020-07120-8

**Published:** 2021-01-06

**Authors:** Xiang Peng, Yani Wang, Xiangwen Xi, Ying Jia, Jiangtian Tian, Bo Yu, Jinwei Tian

**Affiliations:** 1 Department of Cardiology, The Second Affiliated Hospital of Harbin Medical University, 246 Xuefu Road, Nangang District, Harbin, 150086 China; 2 The Key Laboratory of Myocardial Ischemia, Harbin Medical University, Ministry of Education, 246 Xuefu Road, Nangang District, Harbin, 150086 China; 3 Guangxi Key Laboratory of Diabetic Systems Medicine, Guilin Medical University, Guilin, 541000 Guangxi China

**Keywords:** COVID-19, Cytokine storm, Heart failure, Treatment

## Abstract

The coronavirus disease 19 (COVID-19) pandemic poses a serious global threat to human health and the economy. Based on accumulating evidence, its continuous progression involves not only pulmonary injury but also damage to the cardiovascular system due to intertwined pathophysiological risks. As a point of convergence in the pathophysiologic process between COVID-19 and heart failure (HF), cytokine storm induces the progression of COVID-19 in patients presenting pre-existing or new onset myocardial damage and even HF. Cytokine storm, as a trigger of the progression of HF in patients with COVID-19, has become a novel focus to explore therapies for target populations. In this review, we briefly introduce the basis of severe acute respiratory syndrome coronavirus 2 (SARS-CoV-2) and illuminate the mechanism and links among COVID-19, cytokine storm, and HF. Furthermore, we discuss drugs and therapeutic targets for patients with COVID-19 and HF.

## Introduction

Since the first outbreak in December 2019, coronavirus disease 19 (COVID-19), an emerging infection, has rapidly spread and placed an excessively heavy burden on the medical system worldwide, leading to the loss of life. Cytokine storm represents a vital factor causing the progression of both COVID-19 and heart failure (HF). In patients with COVID-19, serum cytokine levels are obviously elevated, which are beneficial to block viral infection at the early stage [1]. However, persistent and excess cytokine infiltration leads to severe tissue damage and even multi-organ failure, including respiratory and circulatory system failure [2]. In HF, cytokines function as important pathogenic factors and vital biomarkers of the progression of various cardiac diseases that eventually lead to HF. In turn, HF also triggers the release of large amounts of cytokines by inducing systemic inflammation [3]. Consequently, knowledge of the basic pathophysiology and immunological process underlying the clinical manifestations of COVID-19, the mechanism underlying cytokine storm induced by severe acute respiratory syndrome coronavirus 2 (SARS-CoV-2), and their contributions to the progression of HF by inducing subsequent immune disorders are urgently needed. In this review, we briefly introduce the basic characteristics of SARS-CoV-2 and illuminate the mechanism underlying the associations among SARS-CoV-2, cytokine storm, and HF, as well as the role of hyperinflammation induced by excess cytokine release in COVID-19 and HF pathophysiologic processes. Furthermore, we discuss drugs and therapeutic targets for patients with COVID-19 who have either a pre-existing cardiac disease or new onset myocardial damage, including HF. We hope that this review will become a valuable reference for the prevention and treatment of HF in patients with COVID-19.

## The Cytokine Storm in Patients with COVID-19

### Virology of COVID-19

SARS-CoV-2, the causative agent of COVID-19, is a spherical, positive single-stranded RNA coronavirus. According to genomic sequencing, SARS-CoV-2 shares 79.5% homology with the sequence of SARS-CoV that caused the outbreak of SARS in 2003 [4]. Compared with the sequences of coronaviruses found in wildlife, SARS-CoV-2 shares 96.2% homology with BatCoV-RaTG13 in bats and approximately 90% homology with coronavirus in pangolins [5, 6]. Consequently, a likely route of viral transmission involves the enlargement of the reproductive scale by SARS-CoV-2 derived from bats infecting one or more intermediate hosts, such as pangolins [7]. In an analysis of the SARS-CoV-2 genome in 103 Chinese patients, SARS-CoV-2 was shown to evolve into type L (~70%) and type S (~30%), and the former type is more invasive and infectious than the ancestral type S [8].

**Fig. 1:**
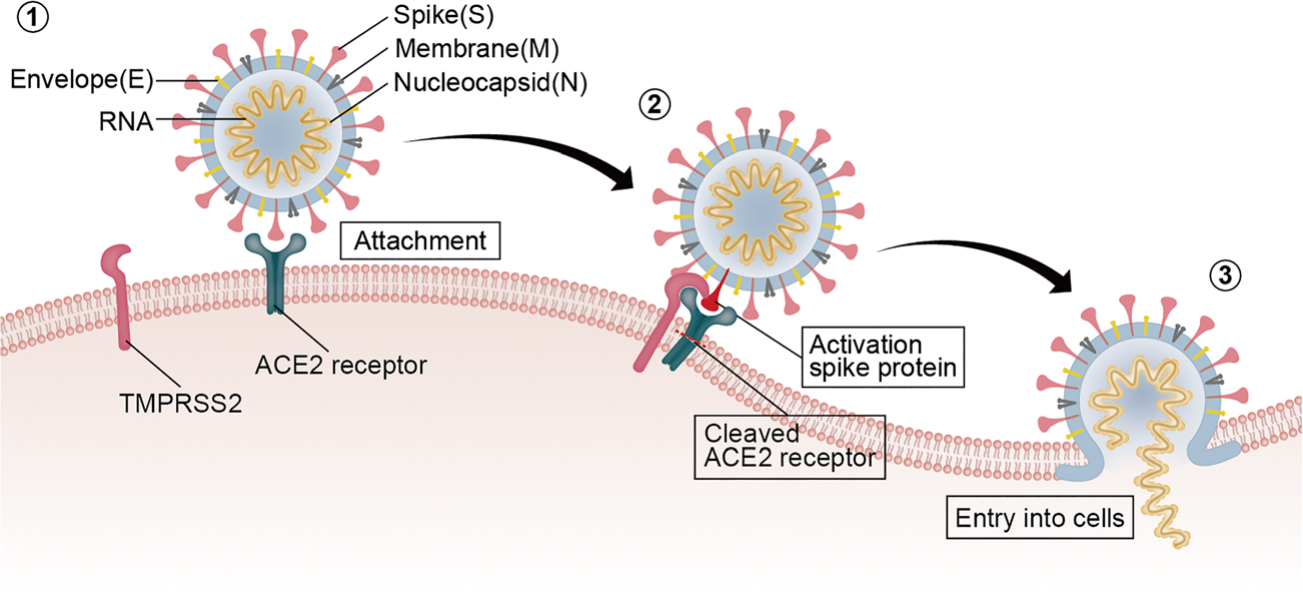
Mechanism by which SARS-CoV-2 enters host cells. (1) SARS-CoV-2 adheres to the surface of the target cell after interacting with ACE2 via the S protein. (2) TMPRSS2 binds and then cleaves ACE2. During the process, the S protein is activated to promote membranous fusion between the virus and host cell. (3) The virus enters the host cell. Abbreviations: ACE2, angiotensin-converting enzyme 2; RNA, ribonucleic acid; SARS-CoV-2, severe acute respiratory syndrome coronavirus-2; TMPRSS2, transmembrane serine protease 2

SARS-CoV-2 consists of structural proteins, such as spike (S), membrane (M), envelope (E), and nucleocapsid (N) proteins, as well as hemagglutinin esterase (HE) proteins present in other viruses [9]. Among these proteins, the S protein is a highly glycosylated protein that forms the trimeric spine on the viral surface to recognize receptors in host cells and mediate membrane fusion. It is a critical factor contributing to host cell infection and plays an important role in regulating the process of virus entry into cells [10] (Fig. 1). The S protein forms the S1 and S2 structural domains, both of which maintain the noncovalent binding of the conformation before fusion. After entrance of the virus into host cells, the S protein is cleaved in intracellular vesicles [11, 12]. The S1 domain receptor-binding domain (RBD) interacts with the angiotensin (Ang)-converting enzyme 2 (ACE2) receptor located in homologous host cells, which is beneficial for the adhesion of the virus to the surface of target cells. Under the synergistic effect of transmembrane protease serine 2 (TMPRSS2), ACE2 cleavage, and S protein priming occur, followed by membrane fusion between the virus and host cells mediated by the S2 subunit, enabling SARS-CoV-2 to enter the host cell compartment [13–15]. In addition, compared with SARS-CoV, SARS-CoV-2 not only displays better binding affinity and binding site stability, but also contains special furin-like cleavage sites on the border between the S1 and S2 subunits [16, 17]. In pulmonary cells that have lost the ability to express cathepsin L at high levels, the S protein is cleaved at these furin-like S1/S2 cleavage sites before internalization, enhancing the effect of TMPRSS2 and internalization of the virus into cells [18], which may partially explain why COVID-19 has greater transmissibility and/or pathogenicity [19, 20].

SARS-CoV-2 endocytosis mediated by ACE2 is an important mechanism of viral invasion [13, 14]. The tissue location and expression levels of ACE2 receptors are associated with the symptoms and dysfunction caused by COVID-19. However, specific variants of genes (e.g., ACE2, TMPRSS2, and interleukin-6 (IL-6)) or the differential expression of related genes may explain the individual differences in the sensitivity and severity of COVID-19 by altering the kinetics of the viral interaction and endocytosis [21]. In addition to pulmonary epithelial cells, ACE2 receptors are also expressed at high levels on the surface of cardiomyocytes and vascular endothelial cells [22], which potentially explains the possible complications of SARS-CoV-2 observed in the cardiovascular system. Moreover, patients with hypertension or cardiovascular disease (CVD) exhibit higher ACE2 expression than healthy individuals, which is assumed to increase their susceptibility to SARS-CoV-2 infection and the likelihood of adverse outcomes in these high-risk populations [23]. In this case, single nucleotide polymorphisms (SNPs) in ACE2 and IL-6 genetic polymorphisms may be related to HF in patients with COVID-19 [21].

### Mechanism of Cytokine Storm Induced by COVID-19

After SARS-CoV-2 infection, alveolar epithelial cells and alveolar macrophages release pathogen-associated molecular patterns (PAMPs) (e.g., viral RNA) and damage-associated molecular patterns (DAMPs) (e.g., ATP and DNA), both of which activates the secretion of chemokines (e.g., C-C motif chemokine ligand (CCL)-2, CCL-3, and CCL-5) and pro-inflammatory cytokines (e.g., IL-1β, IL-6, tumor necrosis factor (TNF), and interferon (IFN)). The recruitment and accumulation of monocytes and T lymphocytes induced by increasing chemokines and pro-inflammatory cytokines control the infection by executing limited inflammation in the infected focus [2, 24]. While inappropriate external stimuli may introduce limited inflammation into the cytokine storm caused by the release of excess cytokines, the latter is considered the main cause of acute respiratory distress syndrome (ARDS) and multiple organ failure [25]. Activated leukocytes (e.g., macrophages and T/B lymphocytes) can produce cytokines [26]. In terms of inflammatory cells, there are a greater number of macrophages in the bronchoalveolar lavage fluid and/or peripheral blood from patients with severe COVID-19 than from patients with mild COVID-19. The enriched macrophages further promote the secretion of cytokines and chemokines, causing local infiltration of excess cytokines [27]. A decrease in the number of peripheral T lymphocytes has been detected in patients with COVID-19, while convalescent serum samples are effective at treating COVID-19 [2]. T lymphocytes are depleted because they are recruited to the infected focus, indicating the severity of COVID-19, and the clinical effects of serum antibodies suggest that the decrease in the number of B cells also participates in the development of COVID-19 [2, 28]. The number of lymphocytes gradually decreases with the progression of the illness [29]. In addition, older patients with fewer lymphocytes are at a higher risk of an increased length of hospitalization and a worse prognosis [30]. Patients with specific cancers, such as hematopoietic or lymphoid malignancies, immunocompromised patients who are undergoing chemotherapy or radiotherapy; patients with immunodeficiency diseases such as AIDS; or patients with systemic autoimmune diseases receiving immunosuppressive treatment are at higher risk of developing serious complications after being diagnosed with COVID-19. Lymphopenia related to COVID-19 is deemed a criterion indicative of the severity of COVID-19, and its biomarkers are used to evaluate the condition of the SARS-CoV-2 infection [31]. However, the mechanism underlying the exhaustion of T lymphocytes remains unclear. A possible explanation is that infection with a low dose of the virus will activate T- and B-cell responses appropriately; however, high-dose viral exposure may delay viral clearance, because inefficient T- and B-cell immunity will induce the cytokine storm to cause severe COVID-19 further [31].

**Fig. 2:**
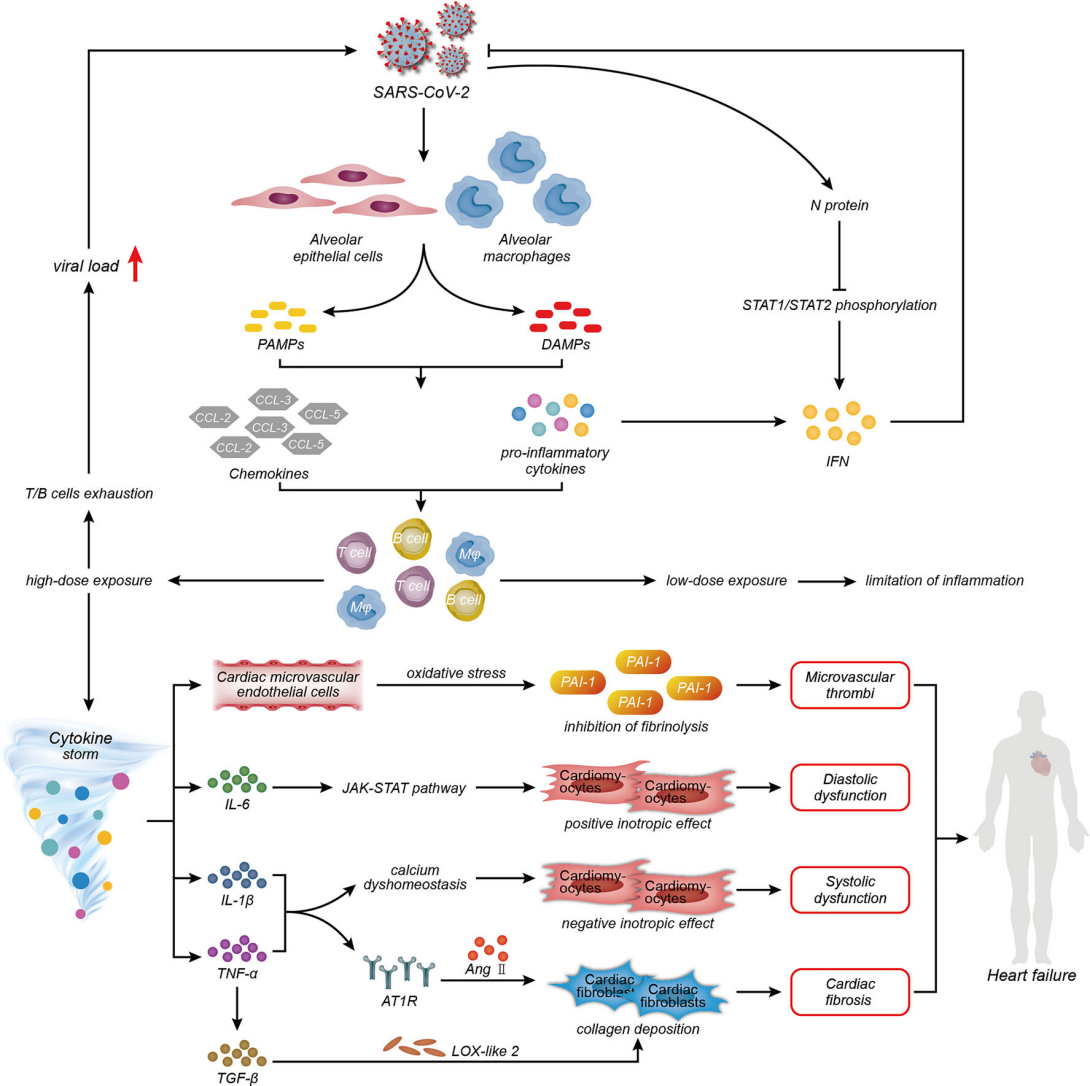
Links among COVID-19, cytokine storms, and HF. After SARS-CoV-2 infection, alveolar epithelial cells and alveolar macrophages release PAMPs and DAMPs, which activate the secretion of CCL-2, CCL-3, and CCL-5 and the pro-inflammatory cytokines IL-1β, IL-6, TNF-α, and IFN to activate and recruit pro-inflammatory cells, including T cells, B cells, and macrophages, to the location of the lesion. Upon exposure to a low dose of SARS-CoV-2, the infection is controlled by limited inflammation, while upon high-dose exposure, T-cell and B-cell exhaustions occur due to the limited inflammation induced by the elevated viral load. Increased IFN levels restrain virus replication, while the SARS-CoV-2 N protein antagonizes signaling through type I IFNs via limiting STAT1 and STAT2 phosphorylation. The persistence of stimulation by SARS-CoV-2 triggers the cytokine storm to secrete large amounts of cytokines that promote the progression of HF. (1) IL-6 induces diastolic dysfunction via positive inotropic effects mediated by the JAK-STAT3 pathway in cardiomyocytes. (2) IL-1β and TNF-α exert a negative inotropic effect by changing intracellular calcium homeostasis in cardiomyocytes to cause systolic dysfunction. (3) IL-1β and TNF-α upregulate AT1R on cardiac fibroblasts and then promote collagen deposition and fibrosis mediated by Ang II. (4) LOX-like 2 expression increases in cardiac fibroblasts via elevated TGF-β induced by TNF-α to promote collagen deposition and cardiac fibrosis. (4) Pro-inflammatory cytokines trigger intravascular oxidative stress in dysfunctional endothelial cells to stimulate the production of PAI-1 and then inhibit fibrinolysis, allowing microvascular thrombi to form. Abbreviations: AT1R, angiotensin type 1 receptors; CCL, chemokine (C-C motif) ligand; COVID-19, coronavirus disease 19; DAMPs, damage-associated molecular patterns; IFN, interferon; IL, interleukin; JAK-STAT3, Janus kinase-signal transducer and activator of transcription pathway; LOX, lysyl oxidase; PAMPs, pathogen-associated molecular patterns; PAI-1, plasminogen activator inhibitor type 1; SARS-CoV-2, severe acute respiratory syndrome coronavirus-2; TGF, transforming growth factor, TNF, tumor necrosis factor

Regarding cytokines, reports have verified that SARS-CoV causes inflammation by promoting viral replication and the release of pyroptosis products after interfering with IFN responses at the early stage [2, 32]. On the one hand, the rapid replication of SARS-CoV induces inflammatory cell infiltration, including macrophages and monocytes, and then inflammatory cells further secrete cytokines and chemokines to recruit more inflammatory cells, representing a self-amplifying cycle that causes more severe disease manifestations through immune dysregulation. In contrast, lymphopenia or an ineffective lymphocyte response caused by infiltration into the lesion induces a persistently elevated viral load that then stimulates excess cytokine release and hyperinflammation. Although SARS-CoV-2 shares 79% sequence homology with SARS-CoV, whether and how the aforementioned mechanism of eliminating IFN responses occurs in individuals infected with SARS-CoV-2 have not been completely clarified. Importantly, SARS-CoV-2 is more efficient at suppressing the IFN-I response than SARS-CoV [33]. According to published reports, patients with COVID-19 who manifest an obvious inflammatory reaction persistently not only exhibit a decrease in interferon activity [34], but also are insufficient to exert the type I IFN responses to control the infection. In addition, they accompany with elevated pro-inflammatory cytokine levels [35]. Similarly, IL-6 levels in these patients gradually increase over time, and the levels in non-survivors are higher than in survivors [36]. Pyroptosis, a type of programmed cell death, associated with a high level of inflammation. IL-1β is a main cytokine released during pyroptosis, and its enhancement can be observed in patients with COVID-19 [29, 37]. Compared with non-intensive care unit (ICU) patients, the serum levels of cytokines, including TNF-α, are higher in ICU patients [29]. Thus, the rapid and vast replication of the virus inhibits the IFN response and induces the depletion of lymphocytes, further causing the infiltration of inflammatory cells and pro-inflammatory cytokines, which may be one cause of severe tissue injury in patients with COVID-19 (Fig. 2).

### The Role of Cytokine Storm in COVID-19

Limited inflammation eliminates the infection and then restores health after the inflammation is resolved in most individuals. However, patients who are elderly or suffer from co-morbidities generally exhibit a dysfunctional immune response that triggers a cytokine storm, allowing widespread inflammation to occur. Subsequently, localized injuries (e.g., diffuse alveolar damage) and systemic injuries (e.g., multi-organ failure) emerge, particularly in the cardiac, hepatic, and renal systems [2]. The multi-organ failure secondary to cytokine storm results in various clinical manifestations due to tissue damage, such as dry/productive cough associated with lung lesions, palpitations related to heart damage, increased aspartate aminotransferase (AST) levels as a marker of hepatic injury, and hematuria related to kidney damage [1, 38]. The most common cause of death in patients with severe COVID-19 may be ARDS with respiratory failure requiring mechanical ventilation and acute cardiac injury with HF [38]. As expected, myocardial damage and circulatory failure caused by increased levels of cytokines have been observed in patients with COVID-19 [39]. The probable explanation for a higher susceptibility to pathogens and a worse ability to control infection in older adults is that a decrease in the number of CD8+ T cells, which is the most prominent marker of immune aging. The severity of COVID-19 increases with age due to a change in the immune response, which is in keeping with the observation of increased odds of in-hospital death due to COVID-19. Furthermore, a high incidence of co-morbidities, such as cardiac dysfunction, has been observed in the elderly [36, 40]. Additionally, age-associated physiological changes (e.g., endothelial dysfunction) and diseases (e.g., obesity and diabetes) induced by inflammation are the main causes of the high incidence of HF in the elderly [41, 42]. In other words, the sensitivity to COVID-19 and HF in elderly patients with severe COVID-19 may be partially due to inflammation mediated by a decrease in the number of T cells. Furthermore, immunomodulatory therapies targeting cytokine storm reduce mortality and improve outcomes in elderly patients with COVID-19; thus, a reasonable hypothesis is that cytokine storm induced by COVID-19 may be one cause of immune aging in elderly patients. Therefore, the excess inflammation caused by cytokine storm is a major risk factor linking the progression and prognosis of COVID-19, particularly in older patients, with co-morbidities. Moreover, subsequent studies should focus on evaluating the curative effect of immunomodulatory therapies on these special patient populations [1]. Interestingly, the gender difference in the susceptibility to COVID-19 may be attributed to the anti-inflammatory and immunomodulatory actions of estradiol and progesterone (P4). High concentrations of 17β-estradiol (E2) block the migration of neutrophils and monocytes by inhibiting the generation of pro-inflammatory cytokines, as well as stimulating B cells to produce antibodies. P4 promotes the production of anti-inflammatory cytokines and inhibits the generation of pro-inflammatory cytokines [43]. Because E2 and P4 not only blunt innate immune inflammation but also promote B-cell responses, clinical trials investigating these hormones are in progress to evaluate the underlying benefits in male patients with COVID-19 (ClinicalTrials.gov identifiers NCT04359329 and NCT04365127).

## HF Induced by Cytokine Storm in Patients with COVID-19

A meta-analysis including eight studies with 46,248 patients with COVID-19 identified hypertension (17 ± 7%, 95% CI 14–22%) and diabetes (8 ± 6%, 95% CI 6–11%) as the most prevalent co-morbidities, followed by cardiovascular diseases (5 ± 4%, 95% CI 4–7%) [1]. Fifty-three patients with COVID-19 and a history of heart diseases and 46 patients without a history of heart diseases were consecutively recruited, and patients with HF accounted for 21% of the total patients and 40% of patients with cardiac diseases [44]. A retrospective case series recruiting 799 moderately to severely ill or critically ill patients with confirmed COVID-19, among which 161 recovered and 113 died, showed that the HF incidence in the patients who died was 49%, a value that is obviously higher than in patients who recovered [45]. HF was deemed one of the most common complications occurring during the exacerbation of COVID-19, and these conditions were more likely to appear in patients with pre-existing cardiac diseases [45]. Thus, the presence of HF in combination with COVID-19, which may occur secondary to a pre-existing cardiac disease or new onset of myocardial damage, is related to an increased mortality risk in patients with COVID-19. Notably, inflammation, which is a factor with dual effects in COVID-19, also interacts with HF to form a chronic vicious cycle by mutually reinforcing the other condition. Therefore, the connections and mechanisms among COVID-19, inflammation and HF are discussed from a pathophysiological perspective in the next section.

### The Progression of HF in Patients with COVID-19

HF manifests various clinical indications in the stages of its progression. The patients who remain in stage A and have risk factors for progression to HF but not structural heart disease can be identified by persistent monitoring of B-type natriuretic peptide (BNP) and/or troponin levels. Cardiac structural damage exists in stage B patients who have not yet presented clinical manifestations, while the manifestations of HF, including dyspnea and peripheral edema, will emerge in stage C, both of which represent a transition period from compensated to decompensated HF and eventually lead to dysfunction. Similarly, we are able to identify the HF stages and progression or prognosis by monitoring dynamic changes in the levels of related biomarkers including neurohormones (e.g., renin–Ang–aldosterone system (RAAS) system), myocyte strain-specific molecules (e.g., BNP and pro-BNP), cardiac injury-induced peptides (e.g., troponin), pro-inflammatory mediators, and oxidative stress components. The intricate pathophysiological mechanism involved must be understood to improve the efficiency of therapy for HF induced by SARS-CoV-2. According to current research data, COVID-19 promotes HF progression and maintains patients in a dangerous condition through complex interactions among multiple risk factors.

1. Direct myocardial injury. SARS-CoV-2 triggers carditis by directly attacking the heart.
2. Inflammation and thrombosis. The release of cytokines induces endothelial dysfunction and circulatory hypercoagulability to promote plaque instability and thrombosis, respectively, subsequently leading to acute coronary syndrome (ACS).
3. Hypoxemia. Lung injury induced by viral attack and pulmonary embolism (PTE) induced by thrombosis enhance hypoxia, which increases the pulmonary vascular resistance by inducing pulmonary vasoconstriction.
4. Downregulation of ACE2. The loss of ACE2 promoted by COVID-19 triggers both vasoconstriction by increasing the expression of Ang and a loss of the protective effects on the heart by decreasing the expression of Ang 1–7.

**Fig. 3:**
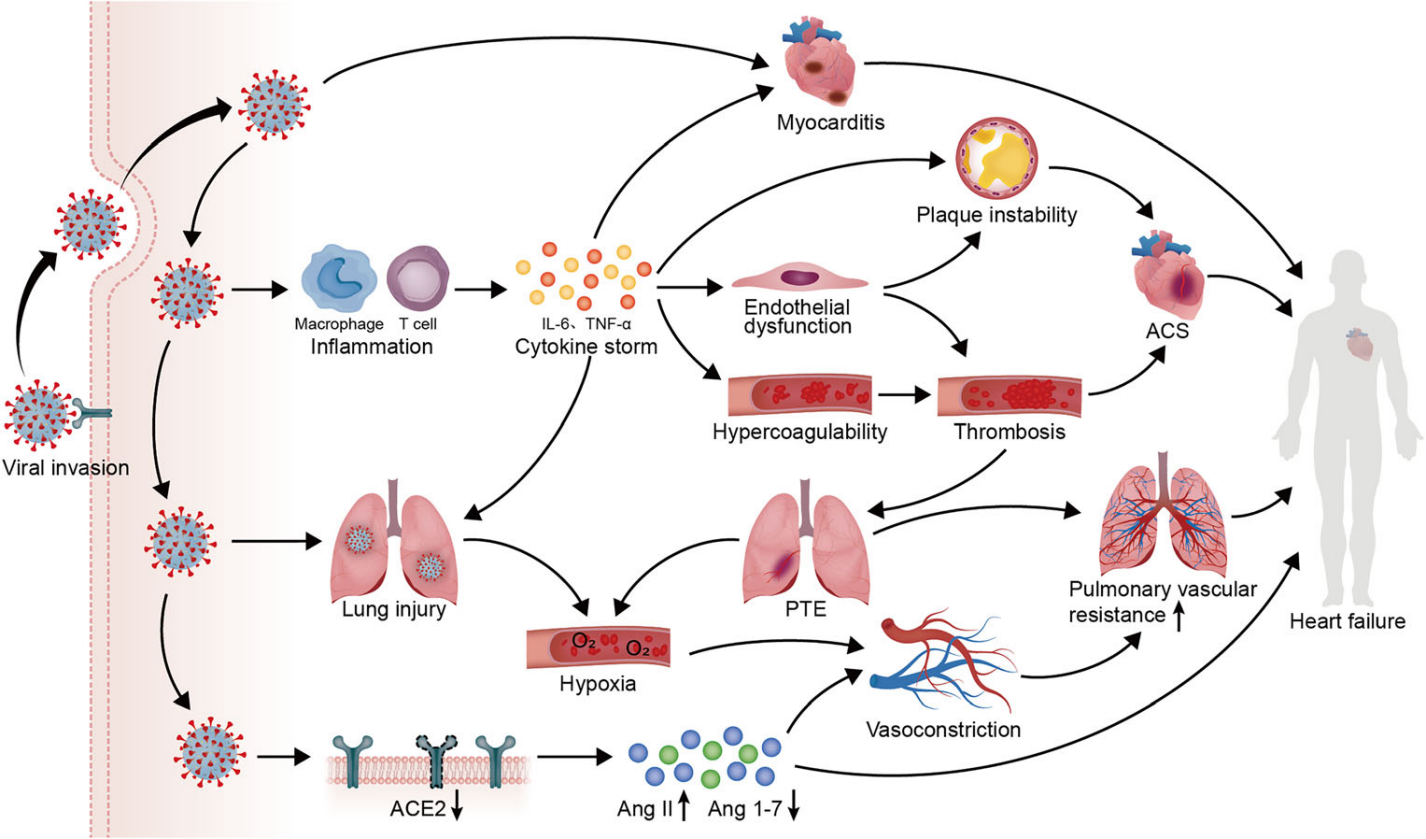
Potential mechanisms of HF deterioration induced by COVID-19. SARS-CoV-2 entry into host cells via ACE2 induces myocardial injury by not only attacking the heart directly to cause myocarditis, but also triggering systemic inflammation and excess immune cell activation to cause a cytokine storm. With the increased release of cytokines, including IL-6 and TNF-α, endothelial cell dysfunction induced by the cytokine storm plays an important role in both plaque instability and circulatory hypercoagulability, promoting thrombosis and ACS. The occurrence of PTE caused by thrombosis worsens the hypoxia induced by lung injury, while pulmonary vasoconstriction under hypoxia increases pulmonary vascular resistance. The loss of ACE2 promoted by COVID-19 triggers both vasoconstriction by increasing the expression of Ang II and a loss of the protective effects on the heart by decreasing the expression of Ang 1–7. In patients with a pre-existing cardiac disease, these mechanisms will work together to promote the progression of HF

Together, these mechanisms will promote the progression of HF in patients with COVID-19 [46] (Fig. 3). Meanwhile, researchers have not clearly determined whether cardiac injury is caused by direct infection of cardiomyocytes or is mainly secondary to lung injury and inflammation [47]. The release of cytokines will participate in the aforementioned structural changes associated with HF, such as cardiac remodeling and endothelial dysfunction regulated by TNF-α, the accumulation of interstitial collagen fibers induced by TNF-α and IL-1, and cardiomyocyte hypertrophy mediated by IL-6. Simultaneously, pro-inflammatory cytokines can promote HF damage induced by oxidative stress [48, 49]. Due to the close correlation between cytokine storm and uncertainty about its regulatory mechanism in COVID-19 and HF, we will emphatically discuss the role of cytokine storm in patients with COVID-19 who develop HF.

### Mechanism of HF Induced by Cytokine Storm

Regarding the mechanism, IL-6 induces diastolic dysfunction by exerting positive inotropic effects mediated by the Janus kinase-signal transducer and activator of transcription 3 (JAK-STAT3) pathway [50–52] and increases cardiomyocyte stiffness by reducing the phosphorylation of titin [53]. IL-1β and TNF-α exert negative inotropic effects by altering intracellular Ca^2+^ homeostasis in cardiomyocytes to cause abnormalities in contractile function [54, 55] and may induce cardiomyocyte pyroptosis and apoptosis, respectively [56, 57]. Notably, the levels of these indicators are elevated in patients with COVID-19 [58]. As expected, myocarditis has been reported in some patients infected with SARS-CoV-2 [59, 60]. ACE2 expressed in the heart exerts anti-inflammatory, anti-fibrotic, anti-oxidant, and vasodilatory effects by withstanding the effect of Ang type 1 receptors (AT1R), while the internalization of SARS-CoV-2 decreases cardiac ACE2 expression [61–63]. IL-1β and TNF-α upregulate AT1R expression in cardiac fibroblasts and favor collagen deposition and fibrosis mediated by Ang II [64], changes that are ameliorated by IL-1β and TNF-α neutralization [61, 65]. Additionally, the rapid decrease in ACE2 expression will limit the generation of Ang II and Ang (Ang 1–7), weakening the cardiac protection provided by the ACE2/Ang 1–7/Mas receptor axis [66]. Meanwhile, the increased level of Ang II promotes phosphorylation of ADAM-17. ADAM-17 not only increases the release of TNF-α to aggravate systemic inflammation, but also accelerates the detachment and loss of ACE2 from the cytomembrane and then impairs the conversion from Ang II to Ang 1–7. Ang II also upregulates the expression of potent neutrophil chemoattractants, including IL-6, or directly stimulates the activation and proliferation of lymphocytes, resulting in persistent inflammation and cytokine storm [67]. Subsequently, these processes constantly amplify the adverse effects of the positive feedback loop and accelerate the progression of HF [68–70]. Failing myocardia express high levels of TNF-α, which has been shown to participate in the development of dilated cardiomyopathy [3]. TNF-α promotes cardiac fibrosis in cardiac fibroblasts. One of the mechanisms is that TNF-α enhances the expression of HF biomarker lysyl oxidase (LOX)-like 2 via elevated transforming growth factor (TGF)-β [71, 72]. Adhesion molecule expression induced by pro-inflammatory cytokines (e.g., IL-1β) in cardiac endothelial cells regulates immune cell adhesion and migration and then accentuates inflammation, while dysfunctional endothelial cells trigger intravascular oxidative stress, which has been observed in patients with SARS-CoV-2 infection [3, 73]. Under oxidative stress, cardiac microvascular endothelial cells cause inhibition of fibrinolysis by stimulating the production of plasminogen activator inhibitor-1 (PAI-1). Subsequently, the persistent existence of microvascular thrombi due to inhibition of fibrinolysis will accelerate the progression of HF [74]. In addition, COVID-19-induced endotheliitis may provide an explanation for the microcirculatory dysfunction in different vascular beds [75]. Briefly, the persistent existence of SARS-CoV-2 stimuli can mediate the cytokine storm and hyperinflammation. Under this state, the progression of HF can be caused through diastolic dysfunction mediated by the Janus kinase-signal transducer and activator of transcription (JAK-STAT) pathway, systolic dysfunction mediated by calcium dyshomeostasis, cardiac fibrosis mediated by elevated TGF-β and AT1R levels, and microvascular thrombi mediated by the inhibition of fibrinolysis (Fig. 2).

### The Links among COVID-19, Inflammation, and HF

As mentioned above, the numbers of T/B lymphocytes are decreased in patients with COVID-19, weakening the ability of these cells to confine and clear SARS-CoV-2. Subsequently, persistent viruses stimulate macrophages to activate and release large amounts of cytokines, leading to hyperinflammation and cytokine storm. Systemic inflammation is the basis of acute or chronic HF and elicits the release of large amounts of cytokines and increased cytokine receptor expression, which potentially represent independent predictors of mortality in patients with advanced HF [76, 77]. Meanwhile, the hemodynamic stress of HF and co-morbidities induce a systemic pro-inflammatory state, and inflammation induced by myocardial infection promote the release of pro-inflammatory cytokines as well [41]. A retrospective single-center case series reported significant correlations between plasma troponin T (TnT) levels and the levels of high-sensitivity C-reactive protein (CRP) and N-terminal fragment of pro-BNP (NT-pro-BNP) in patients with COVID-19 [78]. In addition, the levels of TnT and NT-pro-BNP, which are markers of HF and myocardial damage, exhibited a dynamic escalation in patients who died, although a similar clear change was not observed in survivors [78]. Macrophages may also participate in the progression of HF in patients with COVID-19 by releasing pro-inflammatory cytokines. M1 macrophages secrete pro-inflammatory cytokines, such as IL-6, IL-1β, and TNF-α [79]. Interestingly, cardiomyocyte surfaces present some of TNF-α receptors [80]. The overexpression of TNF-α in mice results in ventricular dilatation, interstitial infiltrates, interstitial fibrosis, and ultimately the occurrence of HF [81]. IL-6 and IL-1β levels are simultaneously increased in patients with HF, which suggests that TNF-α is related to the severity of HF [76]. These indicators can be observed in a later stage of COVID-19, which may be related with a high incidence of cardiovascular events in COVID-19 patients, and the causes of death in these patients are mostly attributed to cardiac arrhythmias and HF [82]. Elevated levels of these indicators of HF, myocardial damage, and inflammation prompted us to speculate that hyperinflammation and cytokine storm elicited by SARS-CoV-2 infection may be among the mechanisms underlying the progression of patients with COVID-19 to HF.

## Implications for Clinical Applications

The associations among COVID-19, cytokine storm, and HF have been described in the aspect of mechanisms, which can provide underlying therapeutic targets for patients with COVID-19 and pre-existing or new onset HF. In this section, we discuss potential targets derived from the inflammatory mechanism and their roles in COVID-19 and myocardial damage, including HF, allowing recommendations to be proposed for target populations.

### Evaluation, Management, and Prognosis of at-Risk Patients

Early identification and timely treatment are beneficial for improving the prognosis of patients with COVID-19 and increasing the utility ratio of strained medical resources. The laboratory index of patients with COVID-19 who develop HF is consistent with acute cardiac injury in retrospective studies [36]; additionally, fatal outcomes in patients with COVID-19, particularly fulminant myocarditis, are consistent with increased levels of inflammatory indicators [39, 83]. Thus, we propose that specific elevated cytokines and appreciably altered inflammatory biomarkers are early warning signals in at-risk patients that may forecast pathogenic conditions and a poor prognosis. The level of CRP in patients with COVID-19 presenting cardiac damage is higher than that in patients without cardiac damage [78]; furthermore, an elevated CRP level is associated with an adverse outcome in patients with HF [84]. The levels of both high-sensitivity troponin I and NT-pro-BNP are increased in patients with COVID-19 presenting with cardiac damage, and troponin has an independent prognostic value for both COVID-19 and HF; however, as a non-independent prognostic marker, NT-pro-BNP is not specific for the diagnosis of concomitant HF in patients with COVID-19 [78, 85]. Additionally, cardiac troponin I levels are substantially increased in patients with severe COVID-19 compared with patients with a mild illness, potentially representing a biomarker to predict the deterioration of the clinical condition of patients with COVID-19 [86]. IL-6, a common characteristic cytokine that participates in cytokine storm, is not only a clinical predictor of mortality in patients with COVID-19 [39], but also an underlying target for decreasing atherosclerosis-related cardiovascular morbidity and mortality [87, 88]. Serum IL-1β and TNF-α levels are obviously elevated in patients with severe COVID-19 [29, 58]. Regarding TNF-α, the proposition that anti-TNF therapy should be initiated at the early and middle stages in patients with COVID-19 to prevent progression has been refuted [89, 90]. Importantly, TNF-α and soluble TNF receptor (TNFR) levels in the circulation are independent predictors of mortality in patients with HF [77]. In addition, IL-1β and its receptor are therapeutic targets in MI and HF [91]; additionally, IL-1β inhibitors improve hyperinflammation and the clinical outcomes of patients with COVID-19 [92, 93]. Because of these increased indexes in patients with COVID-19 presenting with HF, we speculate reasonably that the degree of change and levels of cytokines and related indicators may be related with evaluations of the severity and therapeutic effect, stratified management, and determination of the prognosis of such patients. In addition, the clinical management of chronic conditions should include specific considerations of the underlying cause of a higher risk of death in patients with COVID-19 who have chronic diseases, such as cardiovascular disorders. A novel management protocol that considers cardiovascular diseases and diabetes has been developed, as described below.

1. Telephonic triage by screening the risk factors, symptoms, signs of COVID-19, and body mass index (BMI) should be conducted, and then some recommendations, including self-isolation and close monitoring, should be provided to patients with a BMI of 30.
2. A telemedicine consultation should be provided to all patients to assess the underlying factors influencing diabetes and cardiovascular diseases.
3. Patients who require an immediate medical assessment must visit an emergency department, while others should receive a teleconsultation.
4. Patients who present at the emergency department must undergo diagnostic testing for COVID-19.
5. Related measures must be implemented to protect healthcare workers when patients who require surgery are COVID-19 positive.
6. Patients should ensure that they maintain a proper quarantine after discharge and follow public health guidelines [94].

### Corticosteroids

The use of corticosteroids to treat COVID-19 remains highly controversial. On the one hand, corticosteroids lead to a delay in viral clearance by impairing innate immunity [95, 96]. On the other hand, corticosteroids reverse the tissue damage caused by cytokine storm due to their powerful anti-inflammatory effects [97–99]. According to many clinical studies, corticosteroid treatment is not beneficial to improving tissue injury caused by SARS-CoV-2, which can induce the increasing severity of viral infection, increased mortality rate, and prolonged hospital stays. Given the unsatisfactory clinical outcomes and side effects**,** corticosteroids are not recommended by the current WHO guidelines (released Jan 28, 2020) for the clinical management of severe COVID-19 [100]. However, the powerful anti-inflammatory effects of corticosteroids on cytokine storms should not be neglected in patients with COVID-19. The mortality of patients with influenza A (H1N1) pdm09 viral pneumonia presenting with partial pressure of oxygen (PaO_2_)/fraction of inspired oxygen (FiO_2_) values less than 300 mmHg was reduced by corticosteroid treatment at a low-to-moderate dose (25–150 mg/day methylprednisolone or equivalent) [101]. Because the point-in-time of the peak of the viral load is similar for both SARS-CoV-2 and influenza virus, corticosteroid treatment is worth considering in patients with COVID-19. Notably, methylprednisolone reduced the mortality of patients with COVID-19 presenting ARDS more than 15% and shortened the duration of mechanical ventilation [102]. An early short course of methylprednisolone improved the clinical outcomes and reduced the escalation of care in patients with moderate to severe COVID-19 [97, 102]. Similarly, dexamethasone reduced the mortality of patients with COVID-19 on oxygen therapy or invasive mechanical ventilation, and water-sodium retention was less likely to occur than with the application of methylprednisolone, partially relieving the concern about the use of corticosteroids [96, 103].

Given the urgency of the clinical demands and the uncertainty of the current evidence, the following expert consensus statement regarding corticosteroid treatment of COVID-19 has been proposed: (1) the benefits and harms should be carefully weighed before using corticosteroids; (2) corticosteroids should be used prudently in critically ill patients with novel coronavirus (2019-nCoV) pneumonia; (3) the further use of corticosteroids should be applied with caution in patients with hypoxemia due to underlying diseases or patients who regularly use corticosteroids for chronic diseases; and (4) the dosage should be low to moderate (≤ 0.5–1 mg/kg per day methylprednisolone or equivalent) and the duration should be short (≤ 7 days) [104, 105]. Current guidelines indicate the use of corticosteroids in severe hospitalized COVID-19 patients with ARDS. Furthermore, shorter and relatively lower-dose corticosteroid therapy could be considered [106]. Ciclesonide and mometasone are inhaled corticosteroids (ICSs) that exert an attenuated effect on the replication of SARS-CoV-2 in vitro and are recommended for application in the early stage of COVID-19 [107]. Furthermore, when a patient experiences a cytokine storm, immunosuppression by steroids may be considered. The early administration of a short course of corticosteroids improves the clinical outcome and immune hyper-reactivity in patients with severe COVID-19 [108].

For patients with severe viral myocarditis, the early intravenous administration of adequate corticosteroids is suggested, as this approach exerts an early anti-inflammatory effect and preserves the dying myocardium [109]. Myocarditis may progress to HF with irreversible dilatation if treatment is delayed or not administered [110]. A Cochrane meta-analysis of 719 patients with viral myocarditis revealed that corticosteroids were beneficial treatments for improving cardiac function and that the patients in this group had a higher left ventricular ejection fraction (LVEF) than patients in the control group at the 1- to 3-month follow-up [111]. According to a previous case report, myocarditis induced by the immune checkpoint inhibitor nivolumab was successfully improved by corticosteroid treatment [112]. Similarly, one report has it that corticosteroids followed by tocilizumab and the aldose reductase inhibitor AT-001 may help the recovery of patients with suspected myocarditis in a patient with severe ARDS and suspected myocarditis with ensuing cardiogenic shock caused by COVID-19 [110]. Patients with fulminant myocarditis caused by SARS-CoV-2 have been treated with methylprednisolone combined with i.v. immunoglobulins [110].

Due to drug interactions at the mechanistic level, glucocorticoids not only activate multiple anti-inflammatory genes, but also inhibit the expression of pro-inflammatory cytokines such as TNF-α, IL-1, and IL-6 by activating glucocorticoid receptors. In terms of mechanism, activated glucocorticoid receptors can inhibit the production of pro-inflammatory cytokines by activating relevant transcription factors (e.g., nuclear factor-kappa B (NF-kB) and activator protein-1). Interestingly, activated glucocorticoid receptors will be depleted due to the excess production of activator protein-1 in patients with chronic inflammatory disorders treated with glucocorticoids, that is, the anti-inflammatory effect of glucocorticoids can be disrupted by overly activating relevant transcription factors [113]. Moreover, glucocorticoids inhibit IFN-γ in a concentration-dependent manner, which will reduce the benefit of IFN responses [114]. Given the benefit of IFN responses and the effect of overly activated transcription factors, it is speculated that the use of inhibitors targeting transcription factors may be more appropriate than glucocorticoid therapy in patients with chronic inflammatory disorders. However, evidence is still lacking to support this, and further studies are required.

In conclusion, corticosteroid therapy appears to be a potential COVID-19 treatment that promotes the recovery of myocardial injury, but additional clinical and fundamental experiments aiming to further define whether and how the use of corticosteroids is beneficial to patients with COVID-19 presenting myocardial injury are lacking.

### Interleukin Inhibitors

IL-6 is a pro-inflammatory cytokine that initiates the immune response via the classical cis or trans signaling pathway. The classical cis signaling pathway only exists in immunogenic cells, in which the IL-6 and membrane-bound receptor for IL-6 (mIL-6R) complex activates the JAK-STAT pathway or mitogen-activated protein kinase (MAPK)-NF-kB-IL-6 pathway after binding to the signal transducer glycoprotein 130 (gp130), subsequently triggering the responses of innate and acquired immune system. Meanwhile, IL-6 activates the trans signaling pathway by interacting with soluble receptor for IL-6 (sIL-6R) to induce an extensive pro-inflammatory response in all cells, specifically various endothelial cells [87, 115–117]. Tocilizumab is an mIL-6R and sIL-6R inhibitor that reverses the increases in serum CRP and IL-6 levels in patients with COVID-19. Tocilizumab was approved for treatment in patients with serious COVID-19 presenting elevated IL-6 levels by the Chinese National Medical Products Administration (NMPA) in March 2020, and this treatment has been proposed as a candidate therapy for severe COVID-19 [118, 119]. A retrospective analysis showed that the abnormally increased CRP levels decreased after administering tocilizumab based on conventional treatment. Except that, tocilizumab treatment decreased oxygen intake in 75% of seriously ill patients with COVID-19 and promoted lung lesion opacity absorption in 90.5% of the patients [120]. A meta-analysis showed a 12% decrease in the mortality of patients treated with tocilizumab compared with the control group [121]. Similarly, the latest data from a cohort of 1229 hospitalized patients with COVID-19 suggested that tocilizumab contributed to reducing the death risk, ICU admission rate, or mortality in patients whose baseline CRP levels were greater than 150 mg/L, but had no effect on patients with CRP levels less than 150 mg/L [119]. Regarding safety, the use of tocilizumab as a first-line treatment to inhibit cytokine storm does not cause serious adverse events and improves tachycardia and fever associated with cytokine storm [122–124]. Thus, IL-6R inhibition contributes to the development of novel treatments for COVID-19. In addition, tocilizumab may decrease the burden on the heart during COVID-19 treatment. However, recent research discoveries revealed that both IL-6 and IL-6R blockade may reduce the benefit in patients with COVID-19. The binary complex formed by IL-6 and IL-6R transforms macrophages from the M1 phenotype that promotes inflammation into the anti-inflammatory M2 phenotype [125]. sgp130Fc is a fusion protein of gp130 with the Fc portion of a human immunoglobulin antibody that specifically binds sIL-6R to inhibit intracellular signal transduction and protect tissue from excess inflammatory damage [115, 126, 127]. Therefore, selective blockade of sIL-6R may be a better treatment for patients with COVID-19. Otherwise, JAK-STAT signaling inhibitors, including baricitinib and ruxolitinib, exert potent effects on preventing the progression of a cytokine storm. Baricitinib inhibits endocytosis to limit viral infection at the early stage and decreases IL-6 levels. Consequently, baricitinib can be used at the late stage of COVID-19. Similarly, ruxolitinib reduces the secretion of pro-inflammatory cytokines, such as TNF-α and IL-6, in inflammatory human macrophages. Based on early clinical evidence, ruxolitinib improved the condition of 4 hospitalized patients with COVID-19 who required supplemental oxygen. Additionally, emerging data from a randomized trial analyzing 43 patients with COVID-19 showed a shorter time to an improvement in clinical symptoms and a better survival rate in the therapeutic group treated with ruxolitinib [128]. These IL-6-related signaling targets, including sIL-6R and the JAK-STAT signaling pathway, may represent new approaches to limit hyperinflammation and cytokine storms in patients with COVID-19. Because multiple cytokines exert their effects through these signaling pathways, the inhibitors targeting these signaling pathways may exert broader effects than an inhibitor of a single cytokine.

Interestingly, in a large MI case control study, circulating sIL-6R levels were obviously increased and associated with MI risk and cardiovascular mortality in patients with st - segment elevation myocardialinfarction (STEMI) [129]. A recent study involving a Swedish cohort of 60-year-old men and women revealed a monotone association between an elevated binary/ternary complex ratio and cardiovascular events, leading to the conclusion that IL-6 trans signaling and IL-6:sIL-6R:sgp130 are associated with cardiovascular events [129]. A separate study of patients with stable coronary disease investigating the relationship between circulating levels of sgp130 and the severity of coronary atherosclerosis showed a negative correlation between the serum sgp130 concentration and the risk of cardiovascular events [130]. Consequently, the progression of cardiovascular events may be prevented and treated by inhibitors of IL-6 trans signaling. In conclusion, we reasonably postulate that strategies targeting IL-6R trans signaling may be beneficial for the prevention and treatment of patients with severe COVID-19 and pre-existing cardiac disease or new onset myocardial damage, even HF. Nevertheless, we must consider the situation in which patients with severe COVID-19 who were treated with tocilizumab experienced an increased incidence of secondary infections, and tocilizumab was useless in patients with a hypoinflammatory status or other special conditions [131]. Therefore, further studies are urgently needed to resolve the uncertainties of anti-IL-6 therapy.

IL-1 beta (IL-1β), which is a main activator of IL-6 expression, has attracted our attention in the exploration of COVID-19 therapies [132]. Anakinra, an inhibitor of IL-1β, has been used to treat hyperinflammation at a high dose. A retrospective cohort study of patients with COVID-19 presenting with hyperinflammation (ferritin > 1000 ng/mL and/or d-dimers > 1.5 μg/mL, plus IL-6 < 40 mg/mL) and ARDS (PaO_2_/FiO_2_ < 300) showed that the mortality of patients treated with anakinra was similar to patients administered with tocilizumab. In terms of the curative effect, 55.6% patients administered with anakinra showed a favorable outcome, similar to the tocilizumab-treated matched cohort [133]. In addition, anakinra reduced the return of patients with corticosteroid-dependent and colchicine-resistant recurrent pericarditis [134]. A randomized, placebo-controlled trial showed that canakinumab, a monoclonal antibody for IL-1β, decreased hospitalization for HF in patients with ongoing subclinical inflammation and a history of MI [135]. Thus, anti-inflammatory therapy using an inhibitor of IL-1β may provide a mechanistically distinct approach to the treatment of HF. Namely, the inhibition of IL-1β deserves consideration for patients with COVID-19 presenting with myocardial injury due to its anti-inflammatory activity. Regrettably, adequate clinical data are not available to evaluate the effect of anakinra on patients with COVID-19, but related clinical trials are in progress (CT04330638, NCT04341584, NCT04339712, and NCT04324021) [58].

Because IL-6 trans signaling and IL-1β play vital roles in both COVID-19 and HF, their inhibitors may be effective and novel treatments to prevent the progression of HF in patients with COVID-19.

### Interferons

IFNs are natural broad-spectrum antivirals, and type I IFNs (IFN-α/β) and type III IFNs (IFN-λ) have been applied in the clinic [136, 137]. The immunopathology of SARS-CoV-infected mice was improved by the early administration of type I IFNs [27]. Compared with SARS-CoV, SARS-CoV-2 does not induce an obvious IFN response in infected human lung tissues [138]. The SARS-CoV-2 N protein may explain this phenomenon because it antagonizes type I IFN signaling by limiting STAT1 and STAT2 phosphorylation [139] (Fig. 2). Thus, the interference in the IFN response is more evident in patients with COVID-19. As a result, an uncontrolled viral load leads to damage to various tissues in these patients with impaired IFN response. A multicenter, prospective phase 2 trial showed that a triple combination of IFN beta-1b (IFN β-1b), lopinavir–ritonavir, and ribavirin dominated by IFN β-1b is superior to the control treatment in alleviating symptoms among patients with mild to moderate COVID-19. Moreover, the SARS-CoV-2 load and hospital stays were decreased under such treatment as well [140]. Furthermore, regarding adverse effects, no differences were observed between the two groups. Thus, IFN β-1b is safe for clinical applications [140]. In addition, IFN β-1b improves virus-induced lung fibrosis in mice, and this in vivo experiment provides a theoretical basis for the treatment of patients with acute respiratory distress syndrome caused by SARS-CoV-2 [141]. Consequently, IFN β-1b may be a rational and effective treatment for COVID-19 by launching or improving the antiviral response. Given the peak of the viral load at the time of presentation, the early application of IFNs has been advised to improve injury and prevent the serious complications associated with COVID-19.

In addition to the effect of IFN β on COVID-19, IFN β also plays an important role in HF [142–144]. On the one hand, cardiotropic viruses interfere with the physical function of cardiomyocytes [145, 146]. In contrast, the negative inotropic cytokines induced by the virus may induce hemodynamic deterioration [147]. However, the viral genomes disappear after IFN-β administration, and a decrease in the number of T lymphocytes and an obvious improvement in LV dysfunction have been reported [148]. Thus, IFN β-1b exerts positive effects on virus clearance and the reversion of LV function in patients with myocardial viral persistence. Meanwhile, the partial virus-induced myocardial dysfunction is reversible. Early diagnosis and therapy with IFN-β are important for myocardial dysfunction caused by the virus, even HF. Regarding safety, IFN-β does not exert cardiac-specific adverse effects. Moreover, its clinical benefit may persist due to its antiviral effect [148].

Because type I IFNs induce a systemic inflammatory response to the virus, some scientists have focused on IFN-λ [149, 150]. In contrast to IFN-β, IFN-λ exerts a stronger anti-inflammatory effect on protecting tissues, but lacks the forceful pro-inflammatory effect of type I IFNs [151, 152]. If the infection escapes the control of IFN-λ, the systemic pro-inflammatory response caused by type I IFNs will occur, resulting in further tissue damage due to hyperinflammation [151]. Pegylated or recombinant forms of IFN-λ have been used to inhibit viral replication and block cytokine storms in patients with COVID-19 [152, 153]. IFNs promote ACE2 expression, while the effect of IFNs on blocking SARS-CoV-2 replication may counterbalance the replication-promoting benefits mediated by increased ACE2 expression [150]. Thus, IFN-λ has an anti-virus activity and decreases further damage caused by inflammation. IFN-λ may be inhibited in patients with COVID-19 and then lead to serious complications, including myocardial injury.

In conclusion, IFN-β and IFN-λ may represent effective approaches for the treatment of patients with mild to moderate COVID-19 to prevent or reverse cardiac complications. However, the exact mechanism of IFNs in COVID-19 treatments remains unclear, and the time points associated with the action or treatment of different IFN types still require further exploration.

### RAAS Inhibitors

At present, controversy exists regarding whether patients with COVID-19 should be treated with RAAS inhibitors. On the one hand, ACE2 resists inflammation and tissue damage in the inflammatory environment and may exert protective effects on severe pulmonary complications. RAAS inhibitors also restore reduced ACE2 levels and prevent or even reverse the phenotype of HF. On the other hand, the use of RAAS inhibitors will increase the expression of ACE2, which is important for SARS-CoV-2 to enter host cells. The long-term use of RAAS inhibitors increases the levels of Ang 1–7 [154], which will theoretically increase the sensitivity of host cells to entry and transmission and lead to a higher risk of infection in these patients. Due to the lack of sufficient medical evidence, a clear conclusion regarding the risks and benefits of RAAS inhibitors has not been reached, but the answer will become increasingly clear with ongoing clinical trials.

Recently, a large case control study including 6272 patients with COVID-19 and 30,759 patients as controls did not observe a correlation between the use of RAAS inhibitors and the risk of COVID-19 in a multivariate analysis (angiotensin receptor blockers (ARB), adjusted OR = 0.95 [95% CI 0.86–1.05]; Ang-converting-enzyme inhibitor (ACEI), adjusted OR = 0.96 [95% CI 0.87–1.07]) [155]. Another trial involving 1,128 patients with COVID-19 presenting hypertension showed that the continued use of RAAS inhibitors reduced the risk of death in these patients [156].

In summary, according to the available data, the application of RAAS inhibitors, including ACEIs and ARBs, should be maintained or started in patients with COVID-19 because the benefits of RAAS inhibitors may outweigh the risks. In clinically unstable patients, particularly patients at risk of HF or myocardial infarction, treatment with RAAS inhibitors should not be stopped or switched to treatment with alternative drugs. Because the sudden withdrawal of RAAS inhibitors may lead to an increased early risk of COVID-19-related myocardial injury and accelerate the deterioration of cardiac function, potentially even leading to the development of HF in days to weeks. In brief, withdrawal of RAAS inhibitors would result in an increased risk of cardiovascular death in patients with COVID-19 [103, 157]. Many clinical trials have been launched to investigate the therapeutic value of ACEIs and ARBs (NCT04312009 and NCT04311177). Based on the premise that supplementation of ACE2 will decrease the susceptibility to SARS-CoV-2 and protect the heart while also inhibiting inflammation, a clinical trial of a recombinant form of human ACE2 (rhACE2) Apn01 is ongoing in Europe [158]. Because Ang 1–7 may cause a hyperinflammatory condition, a non-randomized interventional clinical trial designed to evaluate the therapeutic efficacy of Ang 1–7 is ongoing (NCT04375124) [159].

## Conclusions

COVID-19 is a great public health emergency that may cause serious heart damage and result in a worse prognosis and urgently needs to be solved. The cytokine storm caused by SARS-CoV-2 induces systemic hyperinflammation, deteriorating pre-existing cardiac damage or eliciting new onset myocardial injury in patients with COVID-19, which has become a focus for exploring underlying therapeutic targets in COVID-19. In addition, the mechanism underlying the inhibition of cytokine storm may have common targets to inhibit both the progression of COVID-19 and HF, enabling the development of inhibitors of related targets for the treatment of patients with COVID-19 presenting with pre-existing or new onset cardiac disease, even HF. In this review, we sought to reveal the association between COVID-19 and HF in terms of the pathophysiological mechanism, which centers on the cytokine storm. We provided insights into cytokine storm as a trigger of HF progression in patients with COVID-19 and discussed potential anti-inflammatory drugs worthy of further exploration as treatments for COVID-19.

As early warning signals in at-risk patients, clinicians should pay more attention and continuously monitor cytokines and related laboratory indexes. Furthermore, studies are needed to evaluate whether the detection of serum levels of specific cytokines at the early stage contributes to the choice of treatment plans or exerts a curative effect. Hence, large-scale studies are needed to confirm our analysis. We hope that the large number of ongoing prospective, randomized trials will provide more high-quality data and clinical evidence, which will be important for further determining management strategies and optimizing the prognosis of patients with COVID-19.

## Data Availability

Not applicable
